# Supplementary material for: Content-rich biological network constructed by mining PubMed abstracts
Source: BMC Bioinformatics. 2004 Oct 8;5:147. doi: 10.1186/1471-2105-5-147 (PMC528731; doi:10.1186/1471-2105-5-147)
Supplement: Additional File 2 — The original results of the above study (non-essential files are deleted to keep the file size under the limit set by BMC bioinformatics). [file 1471-2105-5-147-S2.bz2 › chilibotAdditionalFile2/dip10/122ID8668348E466/html/MAP2K1_BRAF.html]

 


 **MAP2K1** and **BRAF** 
  
Found 75 abstracts in PubMed, retrieved 10.  
 

 What does Google say? 
 PDF only 
| .edu only 

---

**Interactive relationship** (e.g. stimulation, inhibition, etc)

**Neutral relationship**- In contrast, B Raf  [ **BRAF** ] , a MAPKKK specific for the MEK  [ **MAP2K1** ]  ERK module, binds PA28gamma and a.  Ref: 12650640 Biochem J< MedlineTA>Biochem J,

**Non-interactive relationship** (e.g. studied together, co-existance, homology, etc.)

- Disruption of the Raf MEK  [ **MAP2K1** ]  ERK MAPK kinase pathway, either by RAS or  **BRAF**  mutation, was detected in approximately 62% of all CC and is therefore one of the most frequent defects in cholangiocellular carcinogenesis.  Ref: 12692057 Gut, 2003
- Taken together, our results identify a novel mechanism whereby a Gi o protein coupled receptor inhibits cell proliferation by stimulating ERK signaling via a SHP 1 SHP 2 PI3K Ras Rap1 B Raf  [ **BRAF** ]  MEK  [ **MAP2K1** ]  pathway.  Ref: 12878607 J Biol ChemJ Biol Chem, 2003
